# Effects of edible bird's nest (EBN) on cultured rabbit corneal keratocytes

**DOI:** 10.1186/1472-6882-11-94

**Published:** 2011-10-12

**Authors:** Fadhilah Zainal Abidin, Chua Kien Hui, Ng Sook Luan, Elvy Suhana Mohd Ramli, Lee Ting Hun, Norzana Abd Ghafar

**Affiliations:** 1 Department of Anatomy, Faculty of Medicine, Universiti Kebangsaan Malaysia, Jalan Raja Muda Abdul Aziz, Kuala Lumpur, 50300, Malaysia; 2 Department of Physiology, Faculty of Medicine, Universiti Kebangsaan Malaysia, Jalan Raja Muda Abdul Aziz, Kuala Lumpur, 50300, Malaysia; 3 Institute of Molecular Medical Research, Level 7, Clinical Block, Universiti Kebangsaan Malaysia Medical Centre, Jalan Yaacob Latiff, Bandar Tun Razak, Cheras, Kuala Lumpur 56000, Malaysia; 4 Institute of Bioproduct Development, Universiti Teknologi Malaysia, Skudai, Johor Bharu 81310, Malaysia

## Abstract

**Background:**

There has been no effective treatment or agent that is available for corneal injury in promoting corneal wound healing. Previous studies on edible bird's nest extract (EBN) had reported the presence of hormone-like substance; avian epidermal growth factor that could stimulate cell division and enhance regeneration. This study aimed to investigate the effects of EBN on corneal keratocytes proliferative capacity and phenotypical changes.

**Methods:**

Corneal keratocytes from six New Zealand White Rabbits were isolated and cultured until Passage 1. The proliferative effects of EBN on corneal keratocytes were determined by MTT assay in serum-containing medium (FDS) and serum-free medium (FD). Keratocytes phenotypical changes were morphologically assessed and gene expression of aldehyde dehydrogenase (ALDH), collagen type 1 and lumican were determined through RT-PCR.

**Results:**

The highest cell proliferation was observed when both media were supplemented with 0.05% and 0.1% EBN. Cell proliferation was also consistently higher in FDS compared to FD. Both phase contrast micrographs and gene expression analysis confirmed the corneal keratocytes retained their phenotypes with the addition of EBN.

**Conclusions:**

These results suggested that low concentration of EBN could synergistically induce cell proliferation, especially in serum-containing medium. This could be a novel breakthrough as both cell proliferation and functional maintenance are important during corneal wound healing. The in vitro test is considered as a crucial first step for nutri-pharmaceutical formation of EBN-based eye drops before in vivo application.

## Background

The cornea comprises of three distinct layers: the epithelium, the stroma and the endothelium. Each layer provides specific properties which are crucial to the optimal functionality of the cornea in normal vision while acting as a protective barrier from external environment [1]. The corneal stroma makes up 90% of the corneal volume and is filled with keratocytes bound by extracellular matrix which forms the structural backbone of the cornea [2,3]. Keratocytes are mesenchymal-derived cells of the corneal stroma responsible for the synthesis and maintenance of the extracellular matrix (ECM) components [4]. Normal, quiescent keratocytes residue between collagen lamellae of the corneal stroma as a sparse population of flattened-cells [5], connecting to one other through a network of extensive processes [6]. The keratocytes have low cell turnover with undetectable cell remodelling overtime [5,7,8]. These homeostatic characteristics are important for corneal transparency. During deep injuries in which the epithelial basement membrane is disrupted, these keratocytes will change their morphological characteristics into 'activated' phenotype which resembles the fibroblasts: fusiform-shaped with multiple nucleoli and lack of cytoplasmic granules [9]. The activation of fibroblasts from quiescent keratocytes is also observed in cell culture models which show similar phenotypical changes of keratocytes during wounding. This was done by adding serum into the culture medium and by further passaging these cells, which were initially maintained in a serum-free condition [3,7].

The cornea can be damaged either by limited injury such as abrasions and localized burns or by extensive injury in terms of surface or depth [10]. Following stromal injury, keratocytes are stimulated to either undergo apoptosis or to lose their quiescence and change into activated fibroblasts [11]. The remaining keratocytes then begin to replenish through cell proliferation, followed by cell migration to cover the wounded area [12]. The process of remodelling and tissue repair involves laying down of collagens, proteoglycans and other substances in the wounded area [13]. Unfortunately, topical agents available in the market for treating corneal wounds and ulcers inhibit the healing process because of the presence of preservatives such as benzalkonium chloride (BAK) and polyquartenium-1. These preservatives were used to prevent bacterial contamination and were usually found in multidose preparations [14]. A study by Collin (1986) revealed that BAK should not be used on corneas with abnormal epithelium as it could cause extensive damage involving the mitochondria, other organelles and the outer cell membrane of the corneal stromal cells [15].

The EBN which was derived from swiftlet's saliva has been a renowned heritage delicacy for many generations since the early Chinese dynasties. Although it has a long history as therapeutic herbal medicine and highly-acclaimed for its beauty-rejuvenating applications, very limited research has been done to scientifically prove its medicinal properties. Ancient Chinese literatures described EBN as a precious food because of its unique medicinal property in treating tuberculosis, gastric ulcers and bleeding in the lungs. Greater discoveries on EBN were elucidated by modern science among which was the discovery of the first known avian epidermal growth factor found in purified swiftlet nest extracts [16,17]. EBN was found to have mitogenic effects on human peripheral blood monocytes following stimulation with Concanavalin A and Phytohemagglutinin A [18]. Using EBN and a formulation containing pearl powder, it was shown there was an elevation of DNA synthesis of the T-lymphocytes and circulating immunoglobulin M level in mice, implying the immunoenhancing effects [19]. Guo et al. in 2006 had found out that EBN was able to prevent influenza viruses in vitro via its sialic acid component [20]. Most recently, a study on the effect of EBN on intestinal cells using Caco-2 cell line showed the highest proliferation in commercial EBN compared to the unprocessed EBN obtained from four different zones of Peninsular Malaysia. These findings suggested the presence of growth stimulating components in the commercial EBN which could either be naturally present or because of adulteration [21].

Presently, no study has been done to evaluate the potential value of EBN in promoting corneal cell growth and proliferation that are essential during wound healing. If the EBN can be proven to potentiate cell proliferation, particularly in corneal keratocytes, it will be a major breakthrough in finding another scientific value behind this natural product. Our ultimate aim is to commercialize the EBN-based eye drop as an excellent medical treatment derived from natural sources which could boost the nutri-pharmaceutical industry. Thus, our objective was to identify the ideal dosage of EBN extract for corneal keratocytes using the MTT method as our reference for future studies in in vitro corneal wound healing model. At the same time, we tried to prove the EBN did not alter the characteristics of the corneal keratocytes during cell proliferation.

## Methods

### EBN Preparation

EBN extract coded as EHMG was obtained from Institute of Bioproduct Development, Universiti Teknologi Malaysia (UTM). This extract was prepared under a nondisclosure procedure using an in-house developed method adapted from Oda et al. 1998 [22]. Briefly, the EBN was ground to dust using mortar, pestled and sieved through a 0.4 mm wire mesh to separate the feathers and other impurities. The ground EBN then underwent an aqueous extraction process at a high temperature of 80°C before centrifugation and was then stored at 4°C until used.

### Cell Culture

This study was approved by the Universiti Kebangsaan Malaysia Animal Ethics Committee (project code: UKMAEC Approval Number PP/ANAT/2010/NORZANA/24-AUGUST/321-AUGUST-2010-AUGUST-2011). The corneal tissues were harvested from six New Zealand white rabbits' eyes from the local animal slaughterhouse and were processed using the techniques reported by Norzana et al. (2007) [23].

The stromal tissues were obtained from the corneal tissues after the removal of the loosened epithelial sheets. They were washed thoroughly with phosphate buffered solution (PBS, pH 7.2, Gibco Invitrogen, USA) and each stromal tissue was cut into half. Each piece was digested with 0.3% collagenase type I, incubated at 37°C with intermittent gentle shaking until all the stromal tissues were digested. The stromal cell suspension was centrifuged at 500 × g for 10 minutes; the resultant pellet was washed with phosphate buffered solution (PBS, pH 7.2, Gibco Invitrogen, USA) to remove any residual enzyme and resuspended in the PBS for total cell quantification with haemacytometer (Weber Scientific Int, Ltd. Middlx, England). Cell viability was determined by trypan blue dye (Gibco Invitrogen, USA) exclusion test. Viable stromal cells were seeded in six well-plates (BD Falcon, Franklin Lakes, NJ) with seeding density of 5 × 10^4 ^cell/cm^2 ^in FD medium [F12: DMEM (Dulbecco Modified Eagle's Medium)]. All cultures were maintained in 5% CO_2 _incubator (Jouan, Duguay Trouin, SH) at 37°C under 95% humidity and the media were changed every three days. Once confluent, the primary culture (P0) was trypsinized using 0.05% trypsin-EDTA and counted for total cell number and viability. The culture was then passaged (P1), under the same condition as the primary culture. The morphological features were examined at day 1 using inverted phase contrast microscope (Carl Zeiss, Germany).

### Cell Viability and Proliferation Assay

The 3-(4, 5-dimethylthiazolyl-2)-2, 5-diphenyltetrazolium bromide (MTT) assay was used to assess the effects of EHMG-coded EBN on corneal keratocytes' proliferative capacity and viability. The cells were seeded overnight in 96-well cell culture plate (Cellstar, Greiner Bio-one, Germany) with the seeding density of 5 × 10^3 ^cells cm^-2^. Several concentrations of EHMG-coded EBN using dilution factor of 2 were added in the following day using 2 different media, serum-containing media (FDS) and serum-free media (FD). The cells were incubated at 37°C in a humidified incubator 5% CO_2 _atmosphere for 72 hours until confluence.

Then, each well (including controls) was added with 10 μl MTT solution, mixed and incubated at 37°C in a humidified incubator 5% CO_2 _atmosphere for another 4 hours. Following incubation, 200 μl Dimethylsulfoxide (DMSO) was added to each well to dissolve the purple formazan and the absorbance was measured at 570 nm. The total viable cell number was directly proportional to the level of absorbance produced by the purple formazan precipitate. EBN concentration which provided the highest cell proliferation was used for functional evaluation on keratocytes by mRNA expression assessment.

### Total RNA Extraction and Gene Expression Analyses

Total RNA from cultured keratocytes in A) Serum-containing medium, FDS, B) FDS plus 0.05% EBN, C) Serum-free medium, FD and D) FD plus 0.05% EBN were isolated using TRI Reagent (Molecular Research Center, Cincinnati, USA) according to a previously published protocol. Polyacryl carrier (Molecular Research Center) was added to each extraction to precipitate the total RNA and the extracted RNA pellet was then washed with 75% ethanol and dried before dissolving it in Rnase and Dnase free distilled water (Invitrogen, Carlsbad, USA). The RNA was quantified and the purity was determined by Nanodrop ND-100 spectrophotometer (Wilmington DE, USA) and stored at -80°C before use. Complementary DNA was synthesised from 100 ng of Total RNA with SuperScript III reverse transcriptase (Invitrogen) according to the protocol recommended by the manufacturer. In brief, the protocol conditions were 10 minutes at 23°C for primer annealing, 60 minutes at 50°C for reverse transcription and 5 minutes at 85°C for reaction termination.

The expression of collagen type 1, aldehyde dehydrogenase (ALDH) and lumican were evaluated by two-step reverse transcriptase-polymerase chain reaction (Invitrogen, Carlsbad, USA). Expression of glycerylaldehyde-3-phosphate dehydrogenase (GAPDH) gene was used as internal control to ensure specificity of reaction. The primers (sense and antisense) used in the reaction were designed from NIH Genbank database as shown in Table 1. The two-step RT-PCR reaction was performed using SYBR Green as the indicator in a Bio-Rad iCycler (Bio-Rad, USA). Each reaction mixture consisted of iQ SYBR Supermix, forward and reverse primers (5 μM each), deionised water and 1 μl of cDNA template. The reaction conditions were cycle 1: 94°C for 3 minutes (1 ×), cycle 2: Step 1 94°C for 10-second and Step 2 60°C for 30-second (40 ×), followed by melting curve analysis [24]. The PCR product size was further confirmed with 2% agarose gel (Invitrogen) electrophoresis.

**Table 1:** Description of primers used in RT-PCR for gene expression analyses.

Gene	Accession no:	Primers 5'→ 3'	PCR product (bp)
GAPDH	NM_001082253.1	F: caa cga att tgg cta cag ca R: aaa ctg tga aga ggg gca ga	186

Collagen Type 1	AY633663	F: gcg gag agt act gga ttg acc R: cac acg tgc ttc ttc tcc ttg	163

ALDH	AY503694	F: gag tgg cat gat tca gtg agc R: gag tag tcg tcc cct ctt gga	186

Lumican	AF020292	F: ctg cag ctt acc cac aac aag R: ggt tga agc tca agt cca ggt	160

### Statistical Analysis

All the data were tested for statistical significance using Statistical package for Social Sciences (SPSS) version 18. Values were expressed as mean ± standard error of mean (SEM) and were analyzed using Student's t-test and One-way Analysis of Variance (ANOVA). A p value of less than 0.05 was considered significant.

## Results

### Cell Viability and Proliferative Assay

**Figure 1:**
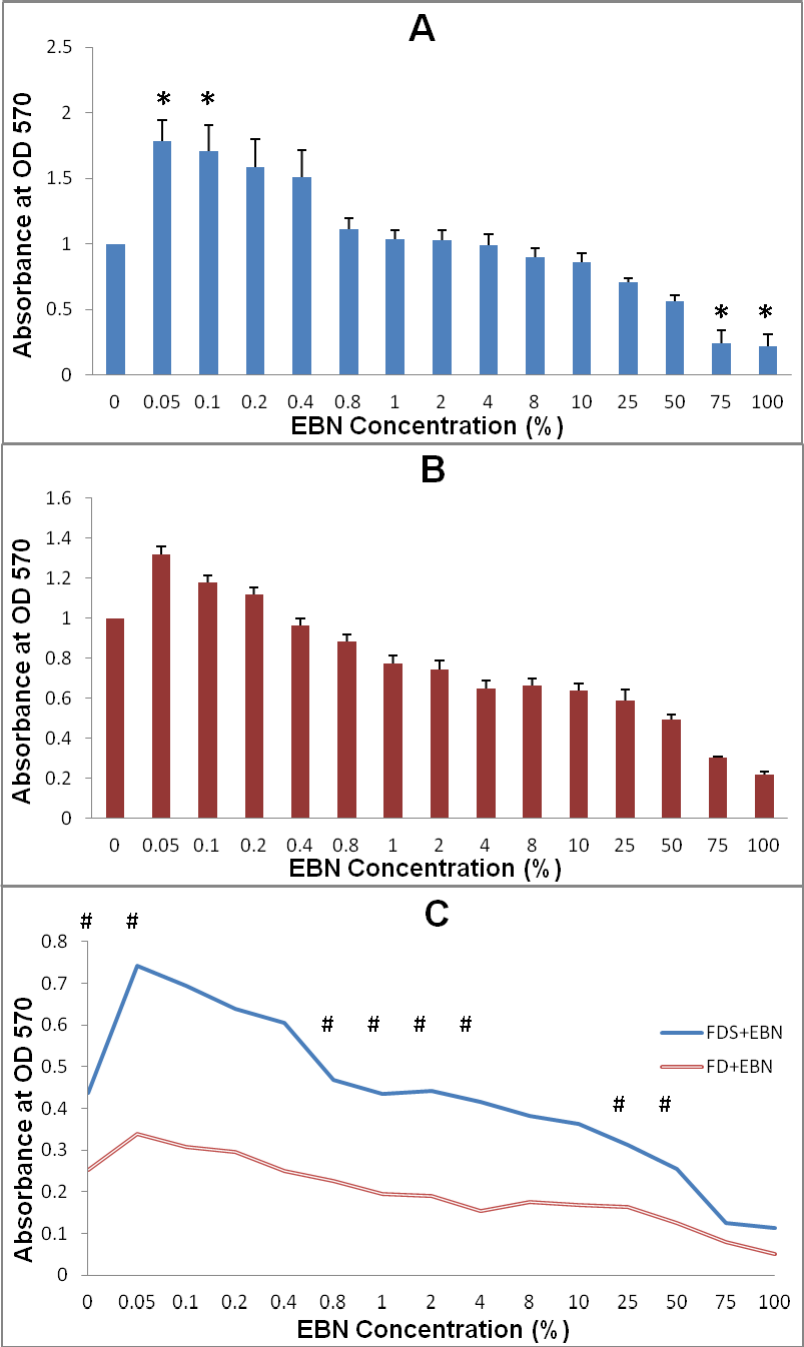
**Viability of corneal keratocytes cultured in serum-containing medium (FDS, 1A), serum-free medium (FD, 1B) supplemented with EBN ranging from 0.05% to 100% and comparison on cell viability between the two groups (1C)**. Significant differences were noted at 0.05%, 0.1%, 75% and 100% in FDS (marked with *). No significant difference was found in keratocytes cultured in FD. Cells cultured in FDS showed significantly higher viability compared to groups in FD. # denotes significant difference (p < 0.05) between groups. Values were tested using Student t-test and expressed as mean ± SEM, n = 6.

The corneal keratocytes cultured in FDS showed higher proliferative potential at 0.05% EBN (p = 0.003) and 0.1% EBN (p = 0.03) [Figure 1A] compared to the FDS alone. Subsequent concentrations of EBN from 0.2% to 50% EBN showed a decreasing pattern in cell proliferation, but these changes were not significant when compared to the control. However, the decrease in cell proliferation was significantly noted at 75% EBN (p = 0.02) and 100% EBN (p = 0.01). This suggested nutrient depreciation from the medium (replacement of medium volume more than 50%) by EBN that leads to a marked reduction in corneal keratocytes proliferation.

On the other hand, corneal keratocytes cultured in FD exhibited the highest proliferative activity when supplemented with 0.05% EBN, followed by 0.1% EBN and 0.2% EBN [Figure 1B], but these results were not significant (p > 0.05) when compared to FD alone. Similar cessation in proliferative capacity of corneal keratocytes was found when tested with a range of EBN concentrations from 0.4% EBN to 100% EBN. However, the values were not significant when compared to the control (p > 0.05).

One important aspect which could also cause higher cell proliferative activity was because of the presence of serum. Corneal keratocytes cultured in FDS showed higher cell proliferative activity compared to FD, with or without the additional EBN [Figure 1C]. The differences were significant among cells cultured in FDS and FD only (p = 0.001), and in media with 0.05% EBN (p = 0.011). There was possibility of mitogenic effect of EBN to corneal keratocytes at this stage, thus promoting higher cell growth. Although the cells cultured with EBN in FDS continuously gave better proliferation compared to those cultured in FD, these differences were only significant between 0.8% to 4% and 25% to 50% EBN (p < 0.05).

### Phase contrast micrographs

**Figure 2:**
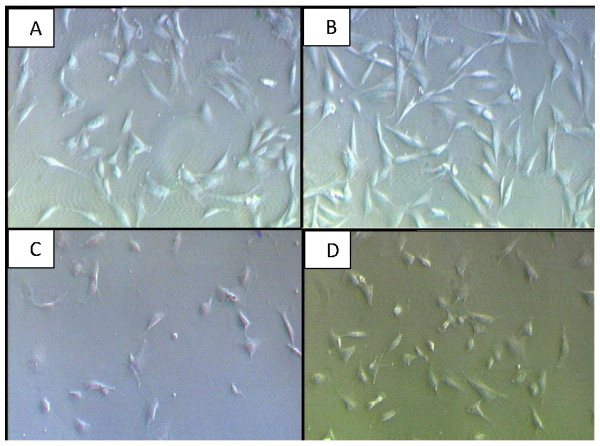
**Phase contrast micrographs under magnification 50 × showing the morphology of corneal keratocytes cultured in different media; A) Serum-containing medium, FDS, B) FDS plus 0.05% EBN, C) Serum-free medium, FD, D) FD plus 0.05% EBN; at Day 1, passage 1**. Supplementation of 0.05% EBN was able to promote higher density of cells.

Microscopic examination of cell morphology on corneal keratocytes showed higher mitotic figures in serum-containing media (Figure 2A &2B) as compared to serum-free media (Figure 2C &2D). This was most apparent in cells cultured in FDS + 0.05% EBN medium (Figure 2B). The corneal keratocytes exhibited typical fibroblast-like feature with fusiform shape in all media, particularly seen in serum-containing media. Similar morphology was observed in corneal keratocytes cultured in serum-free medium (Figure 2C &2D), but with fewer corneal keratocytes. More importantly, corneal keratocytes did not show any abnormal morphological changes when cultured in the EBN-containing media.

### Gene Expression Analysis

**Figure 3:**
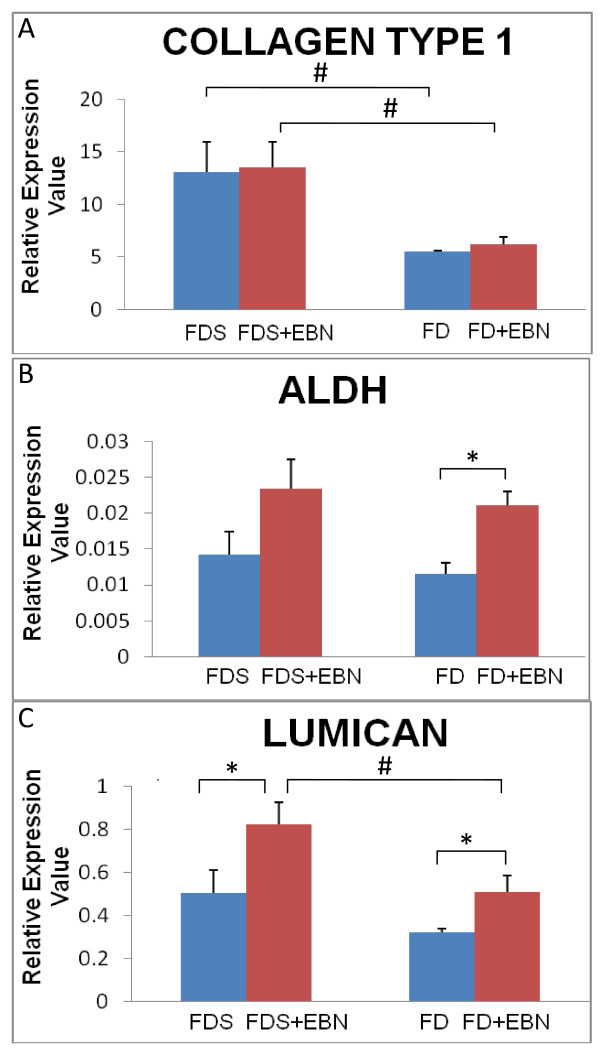
**Quantitative gene expressions of cultured rabbit corneal keratocytes**. The expression values for (A) Collagen Type 1, (B) ALDH and (C) Lumican relative to the expression values of GAPDH as the internal control. Higher expression level was showed in serum-containing groups (FDS, FDS+EBN) compared to serum-free groups (FD, FD+EBN). * denotes significant difference (p < 0.05) in the same group. **# **denotes significant difference (p < 0.05) between groups. Values were tested using Student t-test and expressed as mean ± SEM, n = 6

The real time PCR data showed a higher expression level in the serum-containing groups (with or without supplementation of EBN) compared to serum-free groups (with or without supplementation of EBN). This was probably because of the presence of serum which contains multiple growth factors to promote cells' proliferation and total RNA expression. In this study, the cultured corneal keratocytes were supplemented with 0.05% EBN concentration, since it was the optimal concentration for cell proliferation derived from the MTT assay. In collagen type 1, the expression level was significantly lower in the serum-free groups (with or without EBN) compared to serum-containing groups (p < 0.05) (Figure 3A). For aldehyde dehydrogenase (ALDH), both cells cultured with 0.05% EBN with or without the addition of serum (FDS+EBN and FD+EBN) showed a higher expression level compared to cells cultured in serum-free medium (FD) and serum-containing medium (FDS) alone (Figure 3B) with the significant difference observed in the serum-free groups only (p = 0.01). The expression level of lumican was found to be significantly higher in the EBN-supplemented media in both groups (FD+EBN and FDS+EBN) compared to FD and FDS (p < 0.05) [Figure 3C].

**Figure 4:**
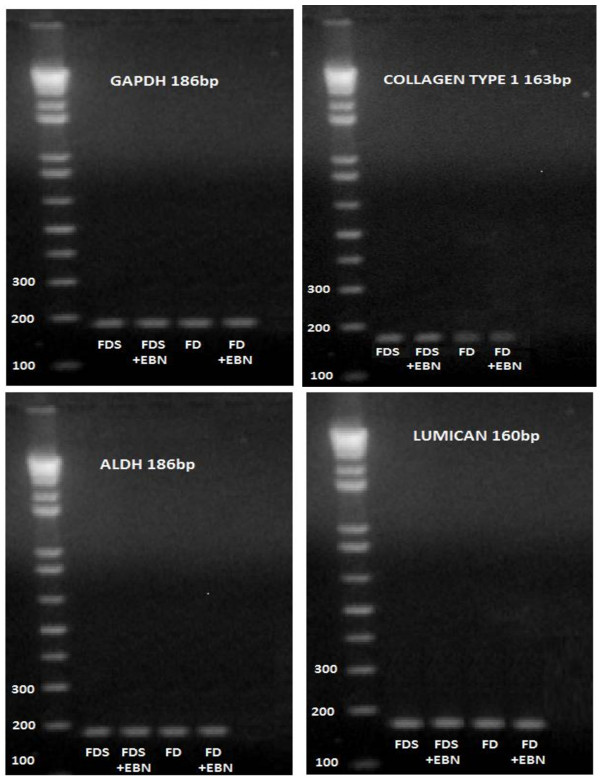
**Gel electrophoresis of cultured rabbit corneal keratocytes phenotypes with GAPDH gene as internal control**.

Gel electrophoresis demonstrated similar intensity of the specific corneal keratocytes associated markers' expressions in FD and FDS with or without EBN. Again, the gene markers used in this study were collagen type 1, aldehyde dehydrogenase (ALDH) and lumican (Figure 4). These indicate the stability of keratocytes phenotype cultured in media supplemented with EBN.

## Discussion

The cornea is exposed to injuries by various ways which includes mechanical insults, infections or following refractive surgical techniques like laser in situ keratomileusis (LASIK) or photorefractive keratectomy. Ideally, when injuries occur to the cornea, the cell layers should regenerate perfectly with the new layers formed resemble the original layers before injury. Unfortunately, most corneal injuries healed by tissue repair were not histologically and physiologically identical with the uninjured corneal tissue. This results in scar tissue formation and opacity of the cornea thus compromising its optical function [2] . Many researchers have proposed different ideas to overcome this problem, for e.g. amniotic membrane application [25-27] or addition of certain growth factors [e.g. epidermal growth factor (EGF), fibroblast growth factors (FGF), transforming growth factors alpha (TGF-α)] [28-31]. However, controlled randomized long-term trials involving large number of patients are still needed to substantiate their clinical importance in promoting effective corneal wound healing [28,31].

The major ingredients of EBN are glycoproteins whereas the major component of carbohydrate in EBN is sialic acid by 9% [32]. Other carbohydrate components include N-acetylgalactosamine (galNac) (7.2%), N-acetylglucosamine (glcNac) (5.3%), galactose (16.9%) and fucose (0.7%) [33]. Amino acids and mineral salts are also found in the EBN, mainly sodium and calcium, with low levels of magnesium, zinc, manganese and iron [34]. Kathan & Weeks in 1969 have found three nonessential amino acids (aspartic acid, glutamic acid and proline) and two essential amino acids (threonine and valine) in EBN. They play an important role in facilitating normal body function such as repairing and providing immunity [35].

We showed that cultured rabbit corneal keratocytes in FDS reached their highest level of cell proliferation with 0.05% and 0.1% EBN (Figure 1A), while those cultured in FD had similar findings with 0.05% EBN alone (Figure 1B). These results were in conformity with the earlier findings of epidermal growth factor (EGF)-like activity using aqueous extract of EBN in stimulating the DNA synthesis of 3T3 fibroblast in vitro [16]. EGF is a 6 kDa polypeptide hormone produced by the salivary and Brunner's glands in the gastrointestinal tract [18] and is also found in human platelets and macrophages [36]. It plays an important role in the normal cellular processes (i.e proliferation, differentiation and development) [18] as well as during inflammation, wound healing and tissue regeneration [37]. Pancholi et al. (1998) has found out that EGF was able to stimulate cell proliferation of cultured keratocytes in a dose-dependent manner [38]. In general, growth factors are effective at very low concentrations while exerting their effects locally [37]. This is in accordance with our findings which showed the highest proliferative activity in keratocytes' culture media supplemented with 0.05% EBN. Concurrently, there was a 70% increase in corneal keratocytes cultured in FDS containing 0.05% EBN compared to only 32% increase of keratocytes cultured in FD containing the same amount of EBN (Figure 2). Culture medium is an important single factor in cell and tissue culture because it supplies all essential nutrients for cell metabolism, growth and proliferation [39,40]. It comprises of all types of soluble molecules - nutrients and salts, hormones and growth factors [41]. Much of these essential nutrients, hormones and growth factors are being supplied by the serum, which is commonly supplemented to the culture medium [42,43]. Hormonal factors in serum potentiate cell growth and proliferation while promoting differentiation [44,45]. The difference in keratocytes proliferation in different media was clearly shown in this study. Similar findings were observed in several hybridoma cell lines in which the specific growth rate and cell productivity had decreased concomitantly with the reduction in serum concentration without medium supplement replacement [46-48]. Besides, EBN may have a synergistic effect in promoting cell proliferation when added into serum-containing medium.

We also observed the corneal keratocytes displayed morphological features of 'activated' fibroblasts when cultured in serum-containing media added with 0.05% EBN. Similar morphological features were observed in keratocytes cultured in serum-free media because of the effect of pasaging. It was important to ensure that EBN besides inducing proliferation of corneal cells was also capable to maintain their phenotypes and functionality by synthesizing and organizing stromal constituents crucial in maintaining corneal transparency. This was further confirmed by the higher functional gene expression of collagen type 1, ALDH and lumican on cultured corneal keratocytes in 0.05% EBN supplemented medium (Figure 4). Collagen Type I is the major structural collagen of the cornea [49]. On the other hand, ALDH catalyzed the oxidation of a wide variety of endogenous and exogenous aldehydes to their corresponding acids, with some ALDH have been identified as corneal crystallins which contribute to the protective and refractive properties of the cornea [50]. The rabbit abundantly expresses ALDHs in its cornea [51]. Lumican is essential for normal cornea morphogenesis during embryonic development and maintenance of corneal topography in adults. Lumican may have additional biological functions, such as modulation of cell migration and epithelium-mesenchyme transition in wound healing and regulating collagen fibrillogenesis [51].

## Conclusions

Much of the work done in this study highlighted the potential use of EBN to treat corneal wound healing. It is indeed the first step in our aim for nutri-pharmaceutical formation of EBN-based eye drop prior to in vivo testing. The results successfully showed the EBN was safe to cornea and more importantly was able to produce cell proliferative activity of corneal keratocytes, optimally at 0.05% concentration. Further studies need to be done to elucidate the specific component of EBN which causes the mitogenic effects to corneal keratocytes. At the same time, there is a continuous need for more scientific researches to confirm the various health claims associated with EBN.

## Competing interests

The authors declare that they have no competing interests.

## Authors' contributions

FZA performed the study, analyzed the data and prepared the manuscript. CKH provided the grants for the study, supervised the work, coordinated the study and corrected the manuscript. NSL performed the gene expression analysis. ESMR supervised the work and corrected the manuscript. LTH participated in the design of the study. NAG provided the grants for the study, supervised the work, coordinated the study, evaluated the data and corrected the manuscript. All authors read and approved the manuscript.

## Acknowledgements

This study was supported by the UKM grant; FF-310-2010. The authors would like to thank the Faculty of Medicine, Universiti Kebangsaan Malaysia (National University of Malaysia) and Institute of Bioproduct Development, Universiti Teknologi Malaysia (UTM).

## Pre-publication history

The pre-publication history for this paper can be accessed here:

http://www.biomedcentral.com/1472-6882/11/94/prepub

## References

[B1] Lu L Reinach PS Kao WWY Corneal epithelial wound healing Exp Biol Med 2001 226 653 664 10.1177/153537020222600711 11444101

[B2] Krachmer JH Mannis MJ Holland EJ Cornea: Fundamentals of cornea and external disease 2004 Mosby-Year Book Publication 183 195

[B3] West-Mays JA Dwivedi DJ The keratocyte: corneal stromal cell with variable repair phenotypes International Journal of Biochemistry & Cell Biology 2006 38 1625 1631 10.1016/j.biocel.2006.03.010 PMC2505273 16675284

[B4] He J Bazan HEP Epidermal growth factor synergism with TGF-β1 via PI-3 Kinase activity in corneal keratocyte differentiation Invest Opthalmol Vis Sci 2008 49 7 2936 2945 10.1167/iovs.07-0900 PMC2614374 18579759

[B5] Fini ME Keratocytes and fibroblast phenotypes in the repairing cornea Prog Retin Eye Res 1999 18 4 529 551 10.1016/s1350-9462(98)00033-0 10217482

[B6] Poole CA Brookes NH Clover GM Keratocyte networks visualized in the living cornea using vital dyes J Cell Sci 1993 106 685 692 10.1242/jcs.106.2.685 8282773

[B7] Davison PF Galbavy EJ Connective tissue remodeling in corneal and scleral wounds Invest Ophthalmol Vis Sci 1986 27 1478 1484 3759366

[B8] Jester JV Petroll WM Cavanagh HD Corneal stromal wound healing in refractive surgery: the role of myofibroblasts Prog Retin Eye Res 1999 18 311 356 10.1016/s1350-9462(98)00021-4 10192516

[B9] Fini ME Streamer BM How the corneal heals: cornea-specific repair mechanisms affecting surgical outcomes Cornea 2005 24 2 11 10.1097/01.ico.0000178743.06340.2c 16227819

[B10] Gnanadoss AS Manual of cornea 2008 Jaypee Bros. Medical Publishes (P) Ltd 62 64

[B11] West-Mays JA Dwivedi DJ The keratocyte: corneal stromal cell with variable repair phenotypes International Journal of Biochemistry & Cell Biology 2006 38 1625 1631 10.1016/j.biocel.2006.03.010 PMC2505273 16675284

[B12] Wilson SE Netto M Ambrosio R Jr Corneal cells: Chatty in development, homeostasis, wound healing, and disease Am J Ophthalmol 2003 136 3 530 536 10.1016/s0002-9394(03)00085-0 12967809

[B13] Dayhaw-Barker P Corneal Wound Healing II: The Process ICLC 1995 22 110 116

[B14] Bartleh JD Ophthalmic Drug Facts 2009 20 Wolters Kluwer Health

[B15] Collin HB Ultra structural changes to corneal stromal cells due to ophthalmic preservatives Acta Ophthalmologica 1986 64 1 72 78 10.1111/j.1755-3768.1986.tb06875.x 3083641

[B16] Kong YC Keung WM Tip TT Ko KK Tsao SW Ng MH Evidence that epidermal growth factor is present in Swiflet's (Collocalia) nest Comparative Biochemistry and Physiology 1987 87 2 221 226 10.1016/0305-0491(87)90133-7 3497769

[B17] Ng MH Chan KH Kong YC Potentiation of mitogenicity response by extracts of swiftlet's (Collocalia) nest Biochem Int 1986 13 521 531 3790144

[B18] Yano S Kondo K Yamaguchi M Richmond G Hutchison M Wakeling A Averbuch S Wadsworth P Distribution and function of EGFR in human tissue and the effect of EGFR tyrosine kinase inhibition Anticancer Res 2003 23 3639 3650 14666659

[B19] Zhang M Wang D Wang J The effects of the Zhenzhu-Yanwo extracts on animal function Pharm Biotech 1994 1 49 51

[B20] Guo C Takahashi T Bukawa W Takahashi N Yagi H Kato K Hidari KI-PJ Miyamoto D Suzuki T Suzuki Y Edible bird's nest extract inhibits influenza virus infection Antiviral Research 2006 70 140 146 10.1016/j.antiviral.2006.02.005 PMC7114130 16581142

[B21] Aswir AR Wan Nazaimoon WM Effects of Edible Bird's Nest on Caco-2 Cell Proliferation Journal of Food Technology 2010 3 126 130

[B22] Oda M Ohta S Suga T Aoki T Study on Food Components: The Structure of N-Linked Asialo Carbohydrate from the Edible Bird's Nest Built by Collocalia fuciphaga Journal of Agricultural and Food Chemistry 1998 46 3047 3053

[B23] Norzana AG Ropilah AR Jemaimah C Chua KH Fauziah O Aminuddin BS Ruszymah BHI Rabbit limbal epithelial cells maintain its stemness in serum-free and feeder layer-free culture system Tissue Engineering and Regenerative Medicine 2007 4 557 565

[B24] Nur Fariha MM Chua KH Tan GC Tan AE Hayati AR Human chorion-derived stem cells: changes in stem cell properties during serial passages Cytotherapy 2011 13 5 582 593 10.3109/14653249.2010.549121 21231803

[B25] Woo HM Kim MS Kweon OK Kim DY Nam TC Kim JH Effects of amniotic membrane on epithelial wound healing and stromal remodeling after excimer laser keratectomy in rabbit cornea Br J Ophthalmol 2001 85 345 349 10.1136/bjo.85.3.345 PMC1723894 11222344

[B26] Shimmura S Shimazaki J Ohashi Y Tsubota K Antiinflammatory effects of amniotic membrane transplantation in ocular surface disorders Cornea 2001 20 408 413 10.1097/00003226-200105000-00015 11333331

[B27] Meller D Pauklin M Thomasen H Westekemper H Steulh K Amniotic membrane transplantation in the human eye Dtsch Arztebl Int 2011 108 14 243 248 10.3238/arztebl.2011.0243 PMC3087122 21547164

[B28] Imanishi J Kamiyama K Iguchi I Kita M Sotozono C Kinoshita S Growth factors: importance in wound healing and maintenance of transparency of the cornea Prog Retin Eye Res 2001 19 1 113 129 10.1016/s1350-9462(99)00007-5 10614683

[B29] Pancholi S Tollu A Khaliq A Foreman D Boulton M The effects of growth factors and conditioned media on the proliferation of human corneal epithelial cells and keratocytes Graefe's Archieve for Clinical and Experimental Ophthalmology 1998 236 1 1 8 10.1007/s004170050034 9457509

[B30] Qin P Kurpakus MA The role of laminin-5 in TGF-alpha/EGF-mediated corneal epithelial cell motility Exp Eye Res 1998 66 5 569 579 10.1006/exer.1997.0455 9628804

[B31] Heather CB Marshall J Growth factors in corneal wound healing following refractive surgery: a review Acta Ophthalmologica 2002 80 3 238 247 10.1034/j.1600-0420.2002.800303.x 12059860

[B32] Colombo JP Garcia-Rodenas C Guesry PR Rey J Potential effects of supplementation with amino acids, choline or sialic acid on cognitive development in young infants Acta Paediatrica 2003 92 42 46 10.1111/j.1651-2227.2003.tb00662.x 12948004

[B33] Dhawan S Kuhad RC Effect of amino acids and vitamins on laccase production by the bird's nest fungus Cyathus bulleri Bioresour Technol 2002 84 35 38 10.1016/s0960-8524(02)00026-3 12137266

[B34] Sam CT Tan PH Lim CH Establishing the authenticity of edible bird's nest ISFM Medicine Scientific Review 1991 3 1 4

[B35] Kathan RII Weeks DI Structure studies of collocalia mucoid. I. Carbohydrate and amino acid composition Arch Biochem Biophys 1969 134 572 576 10.1016/0003-9861(69)90319-1 5354777

[B36] Cotran RS Kumar V Fausto N Robbins SL Abbas AK Robbins & Cotran pathologic basis of disease 2005 Elsevier Saunders

[B37] Andresen JL Ledet T Ehlars N Keratocyte migration and peptide growth factors: the effect of PDGF, bFGF, EGF, IGF-I, aFGF and TGF-β on human keratocyte migration in a collagen gel Curr Eye Res 1997 16 605 613 10.1076/ceyr.16.6.605.5081 9192171

[B38] Pancholi S Tullo A Khaliq A Foreman D The effects of growth factors and conditioned media on the proliferation of human corneal epithelial cells and keratocytes Graefe's Arch Clin Exp Opthalmo 1998 236 1 8 10.1007/s004170050034 9457509

[B39] Nardone RM Cell culture methodology from donor to cell lines BioTechniques 1987 5 122 127

[B40] Butler M Jenkins H Nutritional aspects of the growth of animal cells in culture J Biotechnol 1989 12 97 110

[B41] Freshney RI Culture of Animal Cells: A Manual of Basic Technique 1994 3 NewYork: Wiley-Liss, Inc

[B42] Mizrahi A Lazar A Media for cultivation of animal cells: An Overview Cytotechnology 1988 1 199 214 10.1007/BF00145023 22359116

[B43] Barnes D Sato G Serum-free cell culture: a unifying approach Cell 1980 24 649 655 10.1016/0092-8674(80)90540-1 7460009

[B44] Masters JRW Animal Cell Culture. A Practical Approach 2000 3 Oxford University Press

[B45] Davis JM Basic Cell Culture A Practical Approach 2002 2 Oxford University Press

[B46] Dalili M Ollis DF Transient kinetics of hybridoma growth and monoclonal antibody production m serum-limited cultures Eiotechnol Bioeng 1989 33 984 990 10.1002/bit.260330807 18588012

[B47] Glacken MW Adema E Sinskey AJ Mathematical descriptions of hybridoma culture kinetics. II. The relationship between thiol chemistry and the degradation of serum activity Biotechnol Bioeng 1989 33 440 450 10.1002/bit.260330409 18587935

[B48] Lee GM Huard TK Palsson BO Effect of serum concentration on hybridoma cell growth and monoclonal antibody production at various initial cell densities Hybridonto 1989 3 369 375 10.1089/hyb.1989.8.369 2744789

[B49] Zimmermann DR Treub B Winterhalter KH Witmer R Fischer RW Type VI collagen is a major component of the human cornea FEBS (Fed Euir Biochem Soc) Lett 1986 197 55 58 10.1016/0014-5793(86)80297-6 3512309

[B50] Mahin A Tilly P Elizabeth MA Roger SH Bovine corneal aldehyde dehydrogenase: The major soluble corneal protein with a possible dual protective role for the eye Experimental Eye Research 1990 51 4 419 426 10.1016/0014-4835(90)90154-m 2209753

[B51] Kao WW Liu CY Roles of lumican and keratocan on corneal transparency Glycoconj J 2002 19 4-5 275 285 10.1023/A:1025396316169 12975606

